# Anxiety-Related Adverse Event Clusters After Janssen COVID-19 Vaccination — Five U.S. Mass Vaccination Sites, April 2021

**DOI:** 10.15585/mmwr.mm7018e3

**Published:** 2021-05-07

**Authors:** Anne M. Hause, Julianne Gee, Tara Johnson, Amelia Jazwa, Paige Marquez, Elaine Miller, John Su, Tom T. Shimabukuro, David K. Shay

**Affiliations:** 1 CDC COVID-19 Response Team.

On April 7, 2021, after 5 weeks’ use of the Janssen COVID-19 vaccine under the Food and Drug Administration (FDA) Emergency Use Authorization (EUA), CDC received reports of clusters of anxiety-related events after administration of Janssen COVID-19 vaccine from five mass vaccination sites, all in different states. To further investigate these cases, CDC interviewed vaccination site staff members to gather additional information about the reported events and vaccination site practices. Four of the five sites temporarily closed while an investigation took place. Overall, 64 anxiety-related events, including 17 reports of syncope (fainting), an anxiety-related event, among 8,624 Janssen COVID-19 vaccine recipients, were reported from these sites for vaccines administered during April 7–9. As a follow-up to these interviews, CDC analyzed reports of syncope shortly after receipt of Janssen COVID-19 vaccine to the Vaccine Adverse Event Reporting System (VAERS), the vaccine safety monitoring program managed by CDC and FDA. To compare the occurrence of these events with those reported after receipt of other vaccines, reports of syncopal events after influenza vaccine administered in the 2019–20 influenza season were also reviewed. Syncope after Janssen COVID-19 vaccination was reported to VAERS (8.2 episodes per 100,000 doses). By comparison, after influenza vaccination, the reporting rate of syncope was 0.05 episodes per 100,000 doses. Anxiety-related events can occur after any vaccination. It is important that vaccination providers are aware that anxiety-related adverse events might be reported more frequently after receipt of the Janssen COVID-19 vaccine than after influenza vaccination and observe all COVID-19 vaccine recipients for any adverse reactions for at least 15 minutes after vaccine administration.

CDC interviewed staff members from the five mass COVID-19 vaccination sites that reported anxiety-related adverse event clusters after receipt of Janssen COVID-19 vaccine, focusing on site capacity and layout, vaccination processes, timeline of reported events, and clinical follow-up. Each of the five sites reported all anxiety-related events to VAERS; reports for each event were reviewed by CDC. VAERS is a national passive surveillance system that monitors adverse events after all vaccinations (*1*). VAERS reports are accepted from health care providers, vaccine manufacturers, and the public. Signs and symptoms in VAERS reports are coded using the Medical Dictionary for Regulatory Activities (MedDRA) preferred terms.* VAERS reports are classified as serious if any of the following are reported: hospitalization, prolongation of hospitalization, life-threatening illness, permanent disability, congenital anomaly or birth defect, or death. An anxiety-related event was defined as any of the following occurring in a person during the 15-minute postvaccination observation period at any of the five sites reporting these clusters: tachycardia (rapid heart rate), hyperventilation (rapid breathing), dyspnea (difficulty breathing), chest pain, paresthesia (numbness or tingling), light-headedness, hypotension (low blood pressure), headache, pallor, or syncope (*2*). Six persons who received diphenhydramine or epinephrine at the vaccination visit were excluded because these events might have represented allergic reactions; none was classified as anaphylaxis.

As a follow-up to these interviews, CDC reviewed VAERS reports received during March 2–April 22, 2021, for adverse events associated with receipt of Janssen COVID-19 vaccine doses administered during March 2–April 12. Syncope, a common anxiety-related event reported by the five mass vaccination sites, has specific MedDRA preferred terms (“syncope” and “syncope vasovagal”) and was the focus of this follow-up investigation. Syncopal events that occurred off-site or ≥1 hour after vaccine administration and those in 16 persons who received diphenhydramine or epinephrine were not included. VAERS reports of syncopal events occurring after receipt of any influenza vaccine administered to persons aged ≥18 years during the 2019–20 influenza season (i.e., July 1, 2019–June 30, 2020) served as a comparison, because influenza vaccine is similarly administered as a single dose and is available to all U.S. adults. Reporting rates were calculated using the approximate number of doses of each vaccine administered during the respective analysis periods.^†^ Descriptive analyses of VAERS data were stratified by vaccine type, sex, and age group. These activities were reviewed by CDC and were conducted consistent with applicable federal law and CDC policy.^§^

## Anxiety-Related Adverse Event Clusters

The five mass vaccination sites reported 64 cases of anxiety-related events (Table 1), occurring during April 7–9, 2021; no event met the VAERS classification of serious. The most commonly reported signs and symptoms were light-headedness or dizziness (56%), pallor or diaphoresis (excessive sweating) (31%), syncope (27%), nausea or vomiting (25%), and hypotension (16%). Thirteen (20%) patients informed staff members of a history of fainting associated with receiving injections or needle aversion. Site A reported events during each of 3 days (April 7–9) and did not suspend vaccination; other sites reported multiple events on a single day, after which vaccination at those sites was temporarily suspended. Prevalence of anxiety-related adverse events ranged from 5.2 to 13.5 per 1,000 persons vaccinated. Among the 64 total cases, 39 (61%) occurred in women. Median patient age was 36 years (range = 18–77 years). Most events resolved within 15 minutes with supportive care.^¶^ Thirteen (20%) patients were transported to an emergency department for further medical evaluation; among these, all five for whom follow-up information was available were released from medical care on the same day.

**TABLE 1:** Characteristics of anxiety-related adverse events after receipt of Janssen COVID-19 vaccine (N = 64) — five U.S. mass vaccination sites, April 7–9, 2021

Characteristic	Vaccination site, no. (%)				
	A	B	C*	D	E
Event date	Apr 7–9	Apr 8	Apr 7	Apr 7	Apr 7
First Janssen vaccination event	Y	Y	Y	Y	N
Drive-through site	Y	Y	N	Y	Y
No. vaccinated, total (per day)	3,901 (881; 1,673; 1,347)	2,323	37	593	1,770
No. of cases,^†^ total (per day)	29 (10; 12; 7)	12	4	8	11
Cases per 1,000 vaccinated, total (per day)	7.4 (11.4; 7.2; 5.2)	5.2	10.8	13.5	6.2
Vaccination temporarily suspended	N	Y	Y	Y	Y
**Case characteristic, no. (%)**					
Women	18 (62)	6 (50)	4 (100)	4 (50)	7 (64)
Age range, yrs (median)	23–77 (42)	21–63 (40)	19–33 (20)	25–62 (34)	18–59 (35)
Transported to emergency department^§^	6 (21)	3 (25)	1 (25)	1 (13)	2 (18)
Reported history of anxiety related to needles or medical visits	7 (24)	4 (33)	1 (25)	0 (0)	1 (9)
**Common signs and symptoms**					
Chest pain	3 (10)	0 (0)	1 (25)	0 (0)	0 (0)
Hypotension	3 (10)	3 (25)	0 (0)	2 (25)	2 (18)
Light-headedness or dizziness	19 (66)	4 (33)	3 (75)	3 (38)	7 (64)
Nausea/Vomiting	10 (34)	2 (17)	0 (0)	1 (13)	3 (27)
Pallor or diaphoresis	7 (24)	2 (17)	1 (25)	6 (75)	4 (36)
Seizure-like activity	0 (0)	0 (0)	1 (25)	3 (38)	1 (9)
Syncope	5 (17)	4 (33)	2 (50)	3 (38)	3 (27)
Tachycardia	2 (7)	1 (8)	1 (25)	0 (0)	0 (0)

**Abbreviations:** N = no; Y = yes.

* Site C was located on a college campus that was vaccinating students.

^†^ An anxiety-related adverse event was defined as any of the following occurring in a person during the 15-minute postvaccination observation period at one of the five sites reporting these events: tachycardia, hyperventilation, dyspnea, chest pain, paresthesia, light-headedness, hypotension, headache, pallor, or syncope. Persons with allergic-like symptoms and those who received diphenhydramine or epinephrine were excluded.

^§^ Thirteen patients were transported to an emergency department for further medical evaluation; all five patients with available follow-up information were released later that day.

Four of the five sites (all except site C) offered drive-through vaccination; sites providing drive-through vaccination had previously administered 1,000–4,000 mRNA COVID-19 vaccines per day without reported similar clusters of events. Four of the five sites (all except site E) administered Janssen vaccine for the first time on the day these clusters were reported.

## Reports of Syncope to VAERS

In addition to the anxiety-related events reported by the five mass vaccination sites, review of all VAERS reports containing the MedDRA term “syncope” or “syncope vasovagal” after vaccination with Janssen COVID-19 vaccine during March 2–April 11 identified 653 eligible reports (reports missing information regarding time of vaccine receipt and event were not included) (Table 2). During March and April 2021, among 7.98 million doses of Janssen COVID-19 vaccine administered in the United States, the VAERS reporting rate of syncope after Janssen COVID-19 vaccination was 8.2 per 100,000 doses. Seventeen (3%) of the 653 reports were classified as serious. One hundred twenty-three (19%) reports indicated that the recipient had a history of syncope associated with receiving injections or needle aversion. Among the 653 VAERS reports of syncope, 327 (50%) occurred in women. The median age of persons with syncope after Janssen COVID-19 vaccination was 30 years (range = 18–82 years). The largest proportion of reported syncopal events after Janssen COVID-19 vaccination occurred among persons aged 18–29 years and decreased in increasing age groups.

**TABLE 2:** Reported syncopal events* per 100,000 persons vaccinated and patient demographic characteristics after receipt of Janssen COVID-19 vaccine and influenza vaccine — Vaccine Adverse Events Reporting System, United States, July 1, 2019–April 12, 2021

Characteristic	Vaccine, no. (%)	
	Janssen COVID-19	Influenza
Reporting date	Mar–Apr 2021	Jul 2019–Jun 2020
No. of syncope cases*	653	60
Doses administered	7,980,000	124,000,000
Rate^†^	8.2	0.05
**Sex**		
Female	327 (50)	28 (47)
Male	325 (50)	32 (53)
Missing	1 (0)	0 (—)
**Age group, yrs**		
Median (range)	30 (18–82)	26 (18–88)
18–29	311 (48)	36 (60)
30–39	164 (25)	12 (20)
40–49	77 (12)	4 (7)
49–59	68 (10)	3 (5)
≥60	26 (4)	5 (8)
Missing	7 (1)	0 (0)

* CDC reviewed reports to the Vaccine Adverse Events Reporting System that contained the Medical Dictionary for Regulatory Activities preferred terms “syncope” or “syncope vasovagal” for all Janssen COVID-19 vaccines administered during March 2–April 12, 2021, and any influenza vaccine administered to an adult aged ≥18 years during July 1, 2019–June 30, 2020. Events that occurred off-site or ≥1 hour after vaccine administration and those in persons who received diphenhydramine or epinephrine at the vaccination visit were not included.

^†^ Cases per 100,000 persons vaccinated.

By comparison, 60 reports of syncope after receipt of influenza vaccination were identified during July 1, 2019–June 30, 2020 (0.05 episodes of syncope per 100,000 doses of influenza vaccine administered). Syncopal events after influenza vaccination were reported most frequently among persons aged 18–29 years; the median patient age was 26 years (range = 18–88 years). Among the 60 reports of syncope after influenza vaccine, 15 (25%) indicated that the patient had a history of syncope or needle aversion. Sixteen (27%) reports indicated that the patient had received more than one vaccine immediately before the syncopal episode.

## Discussion

Anxiety-related events, including syncope, can occur immediately after vaccination with any vaccine and might be caused by anxiety about receiving an injection (*3*). Although four of the five mass vaccination sites that reported anxiety-related events temporarily suspended COVID-19 vaccination, none of the reports to VAERS was considered serious. Reports of syncope were approximately 164 times more common after Janssen COVID-19 vaccination (8.2 per 100,000) than after influenza vaccination (0.05 per 100,000).

Approximately one quarter of the syncopal and other anxiety-related events after receipt of Janssen COVID-19 vaccine described in this report occurred in persons who reported a history of similar events after vaccination. Because the Janssen COVID-19 vaccine is administered as a single dose, this vaccine might be a more attractive option for persons who have needle aversion. Therefore, it is possible that some persons seeking Janssen COVID-19 vaccination could be more highly predisposed to anxiety-related events after being vaccinated. The stress of an ongoing pandemic might also increase anxiety surrounding COVID-19 vaccination. In addition, in mass vaccination situations, an anxiety-related event witnessed by others on-site or reported through media coverage might provoke additional anxiety-induced episodes (*4*).

Approximately one half of reports to VAERS of syncope after Janssen COVID-19 vaccination were for persons in the youngest age group (18–29 years) recommended for vaccination. Adolescents have higher rates of syncope after vaccination. For example, a rate of 7.8 syncopal events per 100,000 doses administered was reported after receipt of quadrivalent human papillomavirus vaccine (*5*). Most VAERS reports of syncope are for children aged 11–18 years (62%), followed by adults aged 19–49 years (25%) (*6*). As use of COVID-19 vaccines expands into younger age groups, providers should be aware that younger persons might be more highly predisposed to anxiety-related events after vaccination than are older persons.

The findings in this report are subject to at least three limitations. First, VAERS is a passive surveillance reporting system and subject to underreporting (*1*). VAERS reports are limited by the information provided by the reporter and might be incomplete, although those missing information regarding the time of vaccination or the time of the event were not included. Likewise, some mass vaccination sites had more information available than did others. Second, because the Janssen vaccine is under EUA and health care providers are required to report potentially life-threatening events, a reporting bias might exist. Finally, the Janssen COVID-19 vaccine was not directly compared with other currently available COVID-19 vaccines and instead was compared with influenza vaccine administered during the 2019–20 season. This was because the population that received Janssen vaccine during its introduction differed from that of the mRNA COVID-19 vaccines because of the prioritization (e.g., by age group, occupation, or underlying health condition) of COVID-19 vaccines.** The population receiving Janssen COVID-19 likely differs from the population who received influenza vaccine in during the 2019–20 season; however, the latter is representative of a typical adult population seeking routine vaccination with a single-dose vaccine.

The anxiety-related events described here were reported before reports of thrombosis with thrombocytopenia syndrome (*7*). The Advisory Committee on Immunization Practices reaffirmed its recommendation for the use of Janssen COVID-19 vaccine on April 23, 2021; a warning for rare clotting events, primarily in women aged 18–49 years is now included by FDA in the EUA and provider and patient information sheets (*8*). Anxiety-related events, including syncope, occurring soon after COVID-19 vaccination could raise concern among other vaccine recipients and staff members, particularly in a mass vaccination setting. All COVID-19 vaccine recipients should be observed for at least 15 minutes after vaccination for anxiety-related and other events (e.g., anaphylaxis or immediate allergic reaction) occurring shortly after vaccination.^††^ Increased awareness of anxiety-related events after vaccination will enable vaccination providers to make an informed decision about continuing vaccination.

**Summary**

**What is already known about this topic?**

Syncope and other anxiety-related events can occur after vaccination and have been reported to the Vaccine Adverse Events Reporting System (VAERS) for other vaccines.

**What is added by this report?**

Five mass vaccination sites reported 64 anxiety-related events, including 17 events of syncope (fainting) after receipt of Janssen COVID-19 vaccine. The reporting rates of syncope to VAERS after Janssen COVID-19 and influenza vaccines (2019–20) were 8.2 and 0.05 per 100,000 doses, respectively.

**What are the implications for public health practice?**

Vaccine providers should be aware of anxiety-related events after vaccination and observe all COVID-19 vaccine recipients for any adverse reactions for at least 15 minutes after vaccine administration.

## Footnotes

* Each VAERS report might be assigned more than one MedDRA preferred term. A MedDRA coded event does not indicate a medically confirmed diagnosis. https://www.meddra.org/how-to-use/basics/hierarchy

† Influenza vaccine doses administered during the 2019–20 season were estimated based on coverage estimates. https://www.cdc.gov/flu/fluvaxview/coverage-1920estimates.htm

§ 45 C.F.R. part 46, 21 C.F.R. part 56; 42 U.S.C. Sect. 241(d); 5 U.S.C. Sect. 552a; 44 U.S.C. Sect. 3501 et seq.

¶ Supportive care included placing the person supine and offering hydration and food.

** CDC provided recommendations on who should be vaccinated first because COVID-19 vaccine supply was initially limited. https://www.cdc.gov/coronavirus/2019-ncov/vaccines/recommendations.html

†† Recommendations for observation after COVID-19 vaccine administration are available at https://www.cdc.gov/vaccines/covid-19/info-by-product/clinical-considerations.html.
